# Hydrogen Peroxide Induced Colitis: A Case Report and Literature Review

**DOI:** 10.1155/2017/6432063

**Published:** 2017-12-25

**Authors:** Dushyant Pawar, Anna Calara, Roy Jacob, Nancy Beck, Alan N. Peiris

**Affiliations:** ^1^School of Medicine, Texas Tech University Health Sciences Center, Lubbock, TX, USA; ^2^Department of Radiology, University Medical Center, Lubbock, TX, USA; ^3^Department of Pediatrics, Texas Tech University Health Sciences Center, Lubbock, TX, USA; ^4^Department of Internal Medicine, Texas Tech University Health Sciences Center, Lubbock, TX, USA

## Abstract

Constipation is a common condition. Hydrogen peroxide enemas have rarely been reported as a home remedy for constipation in the pediatric age group. We present a case report and literature review of hydrogen peroxide induced colitis in pediatric siblings, aged 2 years and 9 years. The siblings presented with vomiting and bloody diarrhea an hour following the enema. Physical exam, vital signs, blood, and electrolyte counts were normal, but CT scans showed mucosal thickening of the rectum and colon. Their symptoms resolved after oral intake was curtailed and fluids were replaced intravenously. We discuss existing reports of complications from hydrogen peroxide enemas. Patients may present with abdominal pain and bloody diarrhea. Onset of symptoms varied from minutes to a day and bowel ulceration with necrosis and perforation has occurred, although fatality is rare. Diagnostic tests included computed tomography (CT) scan, sigmoidoscopy, or biopsy. Recovery period ranged from 3 days to 8 months. Public education regarding the dangers of hydrogen peroxide enemas is needed.

## 1. Introduction

Constipation is a common problem, reported in up to 30% of children. Home treatment for constipation includes eating foods high in fiber such as vegetables, citrus fruits, beans, and whole grains. A healthcare provider should be consulted before giving a laxative or enema to children. Internet searches for homemade remedies to treat constipation deliver hydrogen peroxide enema among the results. Hydrogen peroxide has been known to cause colitis and, in a few cases, bowel perforation. The earliest use of hydrogen peroxide enema in the pediatric population is documented by Olim and Ciuti to treat meconium ileus in the newborn [1].

Herein we present an unusual case of two siblings, aged 2 years and 9 years, presenting with bloody diarrhea and vomiting due to chemical colitis following hydrogen peroxide enema. We also present a systematic review of prior such cases. Few such cases have been reported in the pediatric population.

## 2. Case Presentation

Two siblings, a 2-year-old male and a 9-year-old female, had been constipated for 2 days. Patients did not have a family history of gastrointestinal issues and past medical history was unremarkable. The mother of the patients was providing both children with a regular but meat-free soy-based diet of her own volition. However, the children had rock-hard stools that could not be passed. Both patients were not given any prior conventional or homemade remedies. However, following an Internet search, both patients were then given an enema of hydrogen peroxide mixed with warm water. Concentration of hydrogen peroxide used is unknown. Following the enema, the patients passed stools but, an hour later, started vomiting and had bloody diarrhea.

Upon admission, vital signs were normal. Both patients had soft abdomens on exam, which were nontender to deep palpation, nondistended, and no guarding was noted. Physical exam did not raise concerns for perforations and the patients denied abdominal pain. Complete blood count and metabolic panel for both patients were within normal limits.

Abdominal CT for the patient of age 2 years showed mucosal thickening of the rectum and sigmoid colon with no free air. The patient aged 9 years had a more extensive mucosal thickening of the rectum, sigmoid, and descending colon but showed no perforation or free fluid. Both patients were admitted to the pediatric intensive care unit, had their oral intake withheld, and were given IV fluids. They showed clinical improvement the same day and were then transferred to the pediatric ward. Both patients were discharged 24 hours later with resolution of emesis and bloody stool. They were prescribed polyethylene glycol laxative, 17 grams, daily for constipation.

## 3. Discussion

We found 21 published cases related to hydrogen peroxide enema. Prior cases show the patient population as mostly older adults with constipation from secondary causes. To our knowledge, this is the first case of colitis due to hydrogen peroxide enema in siblings in the pediatric age group. The most recent case report was published in 2016 [16]. Cases related to oral ingestion of hydrogen peroxide as well as those associated with contamination during endoscopic examinations were excluded.

In 11 of the 21 cases, enema was self-administered; in the rest, enema was administered under physician supervision. The enema was administered to treat constipation, except for one case where the patient used it to treat prostate cancer. The enema concentrations varied and are listed in Table 1. Following the enema, patients were able to relieve their constipation but then presented with symptoms of abdominal pain/tenderness, bloody diarrhea, fever, tenesmus, leukocytosis, and/or tachycardia [17]. Endoscopy findings included mucosal friability, exudates, ulceration, necrosis, and/or perforation of the distal colon or rectum (Table 1) [17]. The onset of bloody diarrhea after administration of hydrogen peroxide enema varied from a few minutes to an entire day following the enema. Diagnostic tests varied with some combination of CT scan (4 cases), sigmoidoscopy (11 cases), or biopsy (4 cases). In our case, diagnosis was made solely using abdominal CT scan and patient history, without relying on additional sigmoidoscopy and biopsy findings because the temporal relationship of presenting symptoms following the enema favored the diagnosis of hydrogen peroxide induced colitis.

Recovery period ranged from 3 days to 8 months, with mostly uneventful gradual recovery. Recommended treatment included bowel rest, fluid resuscitation, and broad spectrum antibiotics, NSAIDs, or corticosteroids [17]. Depending on the extent of injury, most patients recovered after conservative medical therapy. However, serious consequences included death due to idiopathic hemolytic reaction following plasma transfusion to correct blood loss [4], portal vein embolism [12], and colonic rupture [3]. A summary comparing and contrasting diagnostic tests findings, medications prescribed, and complications of hydrogen peroxide induced colitis are documented in Table 1.

Hydrogen peroxide is available over the counter in concentrations of 3% and a “food grade” variety of 35%. Although it has a warning label stating “For external use only,” a study by the National Survey of Consumers and Health Professionals found that only 7% of consumers read usage warnings. Hence, public education on the dangers of hydrogen peroxide enema may be needed. It may be effective since patients tend to follow the advice of a competent physician over information obtained from the Internet [18]. Hydrogen peroxide has also been used for other indications like enlarged prostate and cancer of prostate [7]. Therefore, patients with unexplained colitis or proctitis may be queried about use of hydrogen peroxide enema.

Finally, the siblings followed a meat-free soy-based diet, and although soy protein has been known to be an allergen, we did not find an association between soy protein and constipation.

## Figures and Tables

**Table 1:** 

Author and year	Patient age	Presenting history	Complications	Iatrogenic	Concentration used	Symptom onset	Treatment/recovery period
Pumphrey (1951) [2]	50 years	Peptic ulcer. Constipation 6 d prior	Abdominal soreness, severe tenesmus, bloody mucus. Fever 103 F. Bowel mucosa covered with gray tenacious membrane, ulcerated, purulent exudate up to 24 cm from dentate line	Yes	2 : 1 hydrogen peroxide & water	Unknown	Granulation tissue cauterized with silver nitrate. Recovery: 8 m

Pumphrey (1951) [2]	76 years	Chronic constipation	Developed bloody mucus stools every 30–60 mins. Ulcerative proctosigmoiditis up to 24 cm	No (self)	Full strength hydrogen peroxide	30 mins	1 gm sulfasalazine qid, rectal instillations of warm oil, 1 teaspoon of psyllium seed oral bid. Recovery: 3 w

Olim and Ciuti (1954) [1]	2 days	Meconium ileus due to pancreatic cystic fibrosis	None: meconium evacuated	Yes	1 : 3 3% hydrogen peroxide & water, via enterotomy		

Ludington et al. (1958) [3]	62 years	Peptic ulcer	Abd pain, rebound tenderness, left lower quadrant mild rigidity, decreased sensation on right side. Fever 103 F 2 seromuscular tears along mesenteric borders of sigmoid and transverse colon	Yes	125 cc hydrogen peroxide in a liter of warm water, retained for 15 mins	Within hours	Laparotomy for seromuscular tears, gangrenous mucosa 4 cm dia. excised and repaired. Recovery: uneventful

Sheehan and Brynjolfsson (1960) [4]	41 years	Duodenal ulcer, constipation	Severe abd pain, rectal bleeding, vomiting, lost 500 ml of blood. Administered plasma. Died due to hemolytic reaction of unknown causes, followed by anuria, uremia, hemoglobinuric nephrosis	No	Unknown	Immediate	Plasma for blood loss. Died due to following complications

Meyer et al. (1981) [5]	22 years	Constipation	Small bloody bowel movements every half hour with tenesmus and lower abd pain. Friable necrotic mucosa to 15 cm. Rectal mucosa improved but erythematous post 5 d	No	10 ml 3% hydrogen peroxide + 30 ml water	30 mins	Parenteral fluids, antibiotics, and rectal steroids. Recovery: mucosa normal at 10 d, asymptomatic at 1 m

Meyer et al. (1981) [5]	47 years	Constipation	Lower abd cramps, tenesmus, rectal bleeding over 24 hours. Diffuse, granular, friable mucosa and discrete ulcerations w yellow/green pseudomembrane. Focal acute ulcerations with edema and congestion of lamina propria	No	Enema of methylene blue dye + 1 oz (29 ml) peroxide in 500 ml water	5 mins	100 units ACTH over 8 hrs and then 30 mg prednisone for 2 wks. Recovery: no bleeding at 3 d, normal mucosa at 21 d

Meyer et al. (1981) [5]	28 years	Constipation	Onset of severe abd cramps, loose bloody bowel movements. Fever 102 F, BP 150/90, pulse 110, RR 14. Diffuse ulcerations at 10 cm; congestion and edema in lamina propria	No	Enema of 150 ml 3% hydrogen peroxide and 60 ml food coloring	Few minutes later	Parenteral ACTH 100 units for 5 days, prednisone 30 mg daily. Recovery: 7 d, asymptomatic at 5 w

Bollen et al. (1998) [6]	13 years	Chronic constipation after being sexually abused	Abd pain, rectal bleeding, no defecation. Hemorrhagic mucosal ulcerations from rectum to splenic angle. 48 hrs, sclerotic lamina propria. Mucosal glands focally destroyed, ischemia, microabscesses with polymorphonuclear leukocytes	Yes	1 : 1 water & 10% hydrogen peroxide	1 hr	No oral feeding for 48 hrs. Gradual complete recovery

Gan and Price (2003) [7]	67 years	Type 2 diabetes, prostate cancer	Profuse diarrhea, initially nonbloody. Later, bloody tenesmus, urgency, soft and nontender abdomen. Normal rectal examination, other than a diffusely hardened prostate. Friable, inflamed mucosa in rectum, several white patches in inflammation area extending to 15 cm. No fibrosis, scarring	No (self)	100 ml to 200 ml 3.5% hydrogen peroxide enema	24 hrs	Recovery: 10 d

Kirrane and Hoffman (2007) [8]	57 years	Abdominal pain, rectal bleeding, tachycardia	Lightheadedness, general weakness, abdominal pain. 12 hours later, multiple episodes of rectal bleeding. Tachycardia (heart rate 110 bpm). Distended abd, discomfort to palpation, no focal tenderness. Distended large bowel, thickened sigmoid colon consistent with colitis. No free air or gas embolization	No	30 ml 35% hydrogen peroxide + 750 ml water	Within minutes; bleeding post 12 hrs	Recovery: 3 d Treated with intravenous fluids and oral simethicone

Almalouf et al. (2008) [9]	19 years	2 week abdominal pain from chronic cholecystitis, constipation following hydrocodone administration	Post 1 hr, abd pain, rectal bleeding. Acute abd, friable mucosa, leukocytosis, free retroperitoneal air	Yes	Soap suds with hydrogen peroxide (500 ml, unknown conc)	1 hour	Ceftriaxone, metronidazole. NPO 3 d until white blood cell counts within normal range. Recovery: 7 d

Desai and Orledge (2010) [10]	43 years	Left-sided abdominal pain, hematochezia	Hematochezia every 30 mins, bowel wall thickening from rectum to distal one-third of transverse colon	No	Commercial sodium phosphate/sodium biphosphate + hydrogen peroxide	Unknown	Recovery: 1 d

Kibria et al. (2010) [11]	61 years	Constipation unresponsive to milk of magnesia	Abd pain, bloody stools 1 hr later. Left lower quadrant tenderness. Rectosigmoid wall thickening, inflammation Friable mucosa, discrete ulcerations & yellow/green pseudomembranes up to 40 cm from anus. Focal acute ulcerations, congestion of lamina propria	Yes	Enema with 90 ml each of hydrogen peroxide, sodium phosphate, and docusate sodium	1 hr	Parenteral fluids, levofloxacin, and metronidazole. Recovery: 3 m

Volonte et al. (2010) [12]	31 years	Severe constipation following spinal trauma. 4000 ml/wk glycerol enema for defecation	Severe abdominal pain soon after enema. 3 hr later CT showed extensive gas in portal vein. Resolved 48 hr later	Yes	700 cc hypertonic solution of 5% glycerin and 300 cc of 3% hydrogen peroxide solution	Within hours	Recovery: 3 h

Lim et al. (2011) [13]	49 years	Abdominal pain	Lower abdominal pain with bloody stools	No	Unknown	2 hours	NPO, IV fluids, antibiotics. Recovery: 5 d

Love et al. (2012) [14]	59 years	Hypertension, coronary artery disease, diabetes, chronic kidney disease, constipation	Rectal bleeding, fecal incontinence over 12 hrs. Soft abd with diffuse tenderness on left lateral side. Rectal exam, presacral thickening, diminished anal tone. Colitis extending to 35 cm, edema & hemorrhage in lamina propria with “bubbly” appearance of goblet cells	No	120 ml hydrogen peroxide (1 : 1 3% hydrogen peroxide & water)	Minutes after	Antibiotic and mesalamine enema. Recovery: 2 d

Taş et al. (2011) [15]	27 years	Constipation over 2 yrs	Post 2 hrs, abd pain, bloody diarrhea. Mild lower quadrant abd tenderness, painful digital rectal exam. Friable, granular ulcerated rectal mucosa. Rectal histopath exam, mucosal congestion, hemorrhage, necrosis, lymphatic ductal ectasia	No	200 ml 3% hydrogen peroxide	2 hours	Enema with budesonide (2 mg). Recovery: 3 d
